# Adverse effects associated with Kanamycin, Amikacin, Capreomycin and Bedaquiline -a VigiAccess™ study

**DOI:** 10.4314/ahs.v24i2.8

**Published:** 2024-06

**Authors:** Lisa Singh, Varsha Bangalee, Serisha Ramasir, Lehlohonolo John Mathibe

**Affiliations:** 1 School of Health Science, Discipline of Pharmaceutical Sciences, University of KwaZulu-Natal; 2 Division of Pharmacology (Therapeutics), Health Sciences, University of KwaZulu-Natal

**Keywords:** Kanamycin, Amikacin, Capreomycin, Bedaquiline

## Abstract

**Background:**

Multidrug-resistant tuberculosis (MDR-TB) is a prevalent health burden, both in South Africa and globally. The treatment of MDR-TB is both complex and difficult as multiple drugs have to be used concurrently in order to achieve good treatment outcomes for patients. However, there is a lack in the evidence regarding the incidences of specific adverse effects of these drugs.

**Objective:**

The main aim/objective of this study was to investigate and compare reported specific adverse drug reactions (ADRs) associated with kanamycin, capreomycin, amikacin and bedaquiline in MDR-TB patients.

**Methods:**

Secondary data collected over a period of 12 months were sourced from a public access data base, VigiAccess™, and analysed.

**Results:**

There was a steep increase in adverse drug reactions reported for kanamycin with the main adverse reactions being hypoacusis, deafness and tinnitus cases, along with vomiting, nausea and diarrhoea. With capreomycin, there were increases in asthenia and hypoacusis although the latter showed a plateau after some point. Rash and pruritus increased along with cases of death with amikacin and there were reports of prolonged QT interval in the electrocardiogram of patients on bedaquiline in addition to nausea, vomiting and diarrhoea.

**Conclusion:**

There are many specific adverse effects associated with kanamycin, capreomycin, amikacin and bedaquiline. The number of cases of the specific adverse effects also increased with time. Therefore, VigiAccess™ provides a good platform for reporting and awareness of specific adverse effects associated with MDR-TB therapy. This is a vital stepping stone for further research.

## Introduction

South Africa has one of the greatest tuberculosis (TB) burdens in the world, and even today TB continues to be the most significant cause of infectious disease-related death in the country.^1^,^2^ The possible reasons for this are manifold and are complicated by the fact that TB is often associated with poverty in various resource-strained countries. The countries that are most significantly affected are middle to low income countries. South Africa is one of the eight countries globally that is most severely affected by this infection. The poor living conditions of majority of the South African population are evidence to this statement, along with the delayed presentation of patients with TB symptoms at health care facilities. In rural areas, MDR-TB tuberculosis treatment is not readily available for people that require it.^3^ The perceptions and attitudes of the population in lower-resource settings may contribute to people not seeking medical care regarding tuberculosis which may lead to further complications.^4^ Therefore, primary healthcare doctors play an important role in dispelling false information about adverse drug reactions (ADRs) associated MDR-TB therapy.

The Global TB report by the World Health Organisation (WHO) reports an estimated incidence of 301 000 cases of active tuberculosis cases in South Africa.^5^ TB is treated using anti-bacterials, and as with most anti-bacterials the issue of drug resistance is pertinent. Drug resistant TB is a growing issue globally as reflected by the WHO statistics. *Multidrug-resistant tuberculosis* (MDR-TB) occurs when the Mycobacterium tuberculosis bacteria becomes resistant to the core first-line drugs, rifampicin and isoniazid. The regimen is more complex and is of a longer duration compared to drug sensitive TB. The long duration of treatment for TB together with the severe side effects associated with the drugs adversely impacts patient compliance^6^

It was estimated that 11 000 people in South Africa had developed multi-drug resistant-tuberculosis in 2018 as compared to 10 722 cases of MDR-TB cases that were reported for 2017.^7^

In South Africa, the treatment of drug-sensitive TB involves a fixed-dose combination regimen of four anti-TB medications (i.e., rifampicin, isoniazid, pyrazinamide and ethambutol) taken over 2-month intensive phase. This is followed by a 4-month continuation phase consisting of rifampicin and isoniazid only. 8,9 However, the treatment protocols and recommendations for MDR-TB continues to change due to the latest evidence on efficacy and safety of currently-used drugs as well as recent discovery novel medicines. In South Africa the standardized MDR-TB treatment regimen previously comprised of a 24-month regimen (i.e., the six months intensive phase and 18-month continuation phase). This regimen included an injectable aminoglycoside (kanamycin), moxifloxacin, ethionamide, terizidone, ethambutol and pyrazinamide. In circumstances where the standardized regimen cannot be used then a tailor-made regimen would be designed based on individual drug sensitivity tests. The additional drugs that are used in the regimen include levofloxacin, capreomycin and/or para-amino salicylic acid.^10^ However, since 2014 the standardised regimen for the treatment of MDR-TB has been revised to include bedaquiline, linezolid and clofazimine. The revised regimen does not include the use of kanamycin.^11^

The aminoglycosides, kanamycin, amikacin and capreomycin, included in the MDR-TB regimen, are effective against a broad range of bacteria.^12^ Kanamycin is the aminoglycoside of choice to be used in MDR-TB regimens. The use of amikacin in MDR-TB is reserved for use in children and neonates.^13^ Other uses of amikacin other than MDR-TB include drug-induced liver injury, hospital acquired pneumonia and catheter associated urinary tract infection. Capreomycin was not used widely as part of the MDR-TB regimen and is reserved for cases of XDR-TB (tuberculosis that is resistant to isoniazid and rifampicin in the first line regimen and a fluoroquinolone/or a second-line injectable). Aminoglycosides are however, associated with severe side effects which limit their use. The side effects of these drugs often develop as a result of the aminoglycosides being used for an extended period of time or due to the increased dose of the aminoglycosides. The use of aminoglycosides result in toxicity directed to the inner ear as well as the kidneys and aminoglycoside-induced ototoxicity is irreversible.^14^ Ototoxicity is a frequent adverse effect of kanamycin, amikacin and capreomycin. However, capreomycin has been associated with a lower incidence of ototoxicity than amikacin and patients that start with capreomycin therapy tolerate the injectable therapy for a longer period of time, although it resulted in electrolyte disturbances in certain patients.^15^ Subjective language or words such as “frequent”, “occurs commonly” and/or “uncommon” are used often when side effects of kanamycin, amikacin, capreomycin and bedaquiline are reported in the literature, formularies and guidelines.^16^ However, there is a consistency on the extent or incidences of specific side effects such as ototoxicity, hypersensitivity reactions (including urticarial and maculopapular rash), arthralgia, nausea and prolongation of QT interval which are often associated with these drugs. The main aim of this study was to investigate and compare reported incidences of specific adverse drug reactions associated with kanamycin, capreomycin, amikacin and bedaquiline in MDR-TB patients.

## Research methods

### Study design

A secondary research design (secondary quantitative research) was utilised in this study. Data from a public access database (VigiAccess™) was sourced for analysis.

### Data source

VigiAccessTM provides information on adverse drug events reported to the World Health Organisation Programme for International Drug Monitoring (PIDM), Upssala, Sweden. These adverse events are presented according to body systems on the VigiAccess™ website (www.vigiaccess.org). The VigiAccess™ database was searched for the most reported and second most reported adverse effects for kanamycin, amikacin, capreomycin and bedaquiline.

A search was performed monthly and incidences were recorded for 12 months, from July 2020 to June 2021 for the specific adverse effects of the four drugs.

### Data analysis

The information collected on the 18th July 2020 served as the baseline data or the denominator for calculating incidences or accrued ADRs during the period of this study. Statistical significance for categorical variables was tested using the Chi square test at 5% level of significance, using GraphPad Prism 8.4.1 version software. Differences with p-values less than 0.05 were considered significant. Mainly bivariate analyses were used and potential confounding factors were not adjusted for in a manner that would be required in multivariate statistical models. [^L17^]

### Ethical considerations

This study was approved by the Biomedical Research Ethics Committee (UKZN), approval number: BREC/00001870/2020.

## Results

As depicted in Table 1, at baseline, kanamycin was associated with 618 cases of hypoacusis and capreomycin was associated with 107 cases of hypoacusis. Cases of ototoxicity associated with kanamycin was 214 at baseline. Cases of rash numbering 1558 were reported for amikacin at base-line while 425 cases of ECG with prolonged QT interval were reported for bedaquiline.

**Table 1:** Most Reported (Baseline) Cases, on the 18^th^ July 2020

	Kanamycin	Capreomycin
Hypoacusis	618	107
Tinnitus	480	73
Deafness	445	43
Ototoxicity	214	30
Vertigo	112	34
	**Amikacin**	
Rash	1558	
Pruritus	1111	
Rash maculopapular	458	
Urticaria	411	
Rash erythematous	220	
	**Bedaquiline**	
Electrocardiogram QT prolonged	325	
Aspartate aminotransferase increased	45	
Hepatic enzyme increased	39	
Alanine aminotransferase increased	34	
Haemoglobin decreased	34	

With regard to the second-most reported (baseline) cases as shown in Table 2, kanamycin was associated with 433 cases of vomiting, while bedaquiline was associated with 149 cases of vomiting. For amikacin, 161 cases of death were reported, along with 169 cases of the drug ineffective. Capreomycin was associated with 31 cases of asthenia and 15 cases of drug resistance at baseline.

**Table 2:** Second Most Reported (Baseline) Cases, on the 18^th^ July 2020

	Kanamycin	Bedaquiline
Vomiting	433	149
Nausea	271	81
Gastritis	96	15
Abdominal pain	77	23
Diarrhoea	55	45
	**Amikacin**	
Pyrexia	424	
Chills	351	
Chest pain	342	
Drug ineffective	169	
Death	161	
	**Capreomycin**	
Asthenia	31	
Injection site pain	16	
Drug resistance	15	
Pyrexia	14	
Malaise	12	

**Figure 1:**
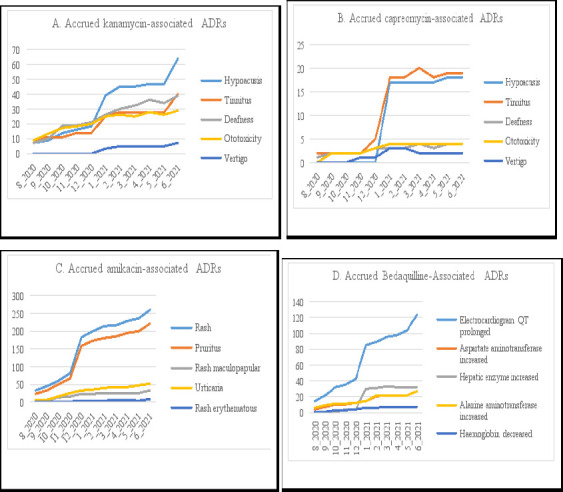
Monthly most commonly-reported ADRs between August 2022 and June 2021

There was a steep increase in reported number of cases of deafness from baseline to June 2021, with 39 new cases associated with kanamycin, as depicted in Figure 1. The number of hypoacusis cases increased steeply from December 2020 to February 2021, with 27 new cases in this time period and 17 new cases from May 2021 to June 2021, as according to Figure 1A. There was also an increase of 12 new cases of tinnitus from May 2021 to June 2021. In Figure 1B, there was a very steep increase in the quantity of cases of hypoacusis associated with capreomycin from December 2020 to January 2021, with 17 new cases being reported after four months of no increase prior to December. After January 2021, a plateau in the number of cases occurred, with little incease in the number of cases till June 2021. As shown on Figure 1C, cases of rash and pruritus associated with amikacin increased rapidly from baseline to June 2021, with 259 new cases of rash and 222 new cases of pruritus. In figure 1D, there was a dramatic increase in the number of cases of ECG with prolonged QT interval associated with bedaquiline from baseline to June 2021, with a total of 124 new cases reported. The other specific adverse effects associated with bedaquiline did not increase substantially from baseline to June 2021, however, they did display increases in the number of cases.

**Figure 2:**
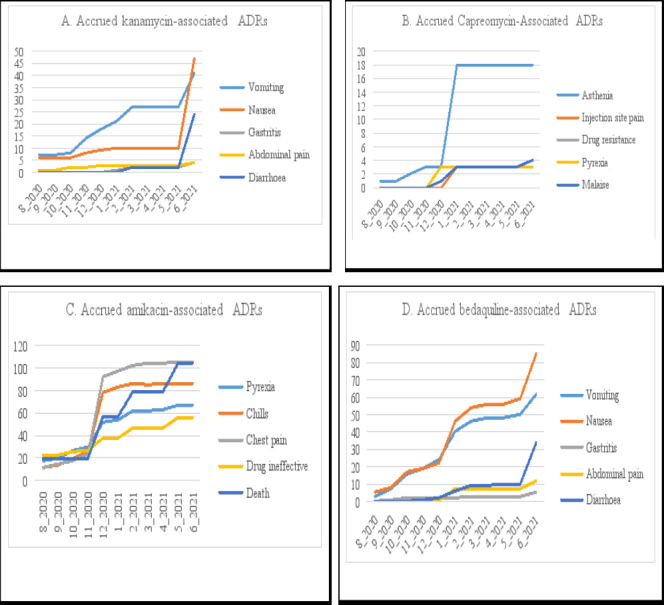
Monthly second most commonly-reported ADRs between August 2022 and June 2021

As depicted in Figure 2A, the reported number of cases of vomiting associated with kanamycin increased steeply from December 2020 to February 2021 with 9 new cases in this period and increased steeply again with 14 new cases from May 2021 to June 2021. There was also a steep increase in the number of new cases of nausea associated with kanamycin from May 2021 to June 2021 with 37 new cases. There were also 22 new cases of diarrhoea from May 2021 to June 2021. There was a dramatic increase in the cases of asthenia associated with capreomycin from December 2020 to January 2021 (Figure 2B), with an increase of 15 new cases in this time period alone (three cases in December 2020 to 18 cases in January 2021). Thereafter, the number of new cases plateaued, with no increase till June 2021. There was no increase in the number of deaths associated with amikacin from baseline to November 2020 (Figure 2C). Thereafter, the number of new cases of death associated with amikacin continued to increase until May 2021 but remained the same till June 2021, with a total of 85 new cases of death from November 2020 to June 2021. As depicted in Figure 2D, there was a steep increase in the number of new cases of nausea associated with bedaquiline from December 2020 to January 2021, with 24 new cases. There was also another steep increase of 26 new cases from May 2021 to June 2021. There was a steep increase in the number of cases of vomiting associated with bedaquiline from December 2020 to January 2021, with 16 new cases and 12 new cases from May 2021 to June 2021. There was a notable increase in the number of new cases of diarrhoea associated with bedaquiline from May 2021 to June 2021, with 24 new cases in this time period. The other specific adverse effects associated with bedaquiline showed little increase in the number of new cases from baseline to June 2021. Bedaquiline was more likely to cause nausea than kanamycin (p = 0.012). Other direct comparisons/differences were not statistically significant.

## Discussion

This was the first longitudinal study to successfully utilise VigiAccessTM data to investigate reported specific ADRs associated with new and old therapy for MDR-TB. Regarding the most reported ADRs, the main findings of this novel study suggest that the number of new cases associated with MDR-TB continue to increase.

Although the five most reported ADRs for kanamycin and capreomycin were similar, the patterns of increase in the cases were different. There was a steady increase in reported cases associated with kanamycin, while there were no or very few new cases associated with capreomycin between December 2020 and June 2021. The possible reasons for the difference in the patterns of increase may be due to kanamycin being an antibiotic that can be used to treat other bacterial infections (other than MDR-TB), while capreomycin is used only to treat MDR-TB.^15^ It is also possible that the use of capreomycin has decreased due to the introduction of new anti-tubercular drugs. Nevertheless, capreomycin was associated with fewer adverse effects cases as compared to kanamycin. This was also reported by Dillard and colleagues 17 who also reported that capreomycin was found to be less ototoxic than the other aminoglycosides.

The five most reported ADRs for amikacin and bedaquiline were different. Therefore, direct comparison was not possible. However, it was worth noting that there were similar patterns regarding rash and pruritus associated with amikacin. New cases of rash and pruritus were increasing tremendously during the period of this study. It is possible that this was due to amikacin being administered as an injection.^18^ Rash and pruritus due to the rash, may have developed at the site of administration.^19^ As these are specific adverse effects, there are no other studies that provided similar outcomes. However, Prasad and collegues^20^ reported that rash, itching and allergic reactions can be caused by anti-tubercular first-line drugs and second-line drugs. It was recently reported that the skin reaction that some patients displayed with amikcin was allergy associated Drug Rash with Eosinophilia and Systemic Symptoms (DRESS) syndrome. 21

Although a pattern of new bedaquiline-induced liver injury cases formed a plateau between January to June 2021, there was a sharp increase in the reported prolongation of QT interval in the electrocardiogram (ECG) incidences for the same period. This indicated that patients were more likely to experience prolonged QT intervals as compared to the other specific adverse drug reactions associated with bedaquiline. This is supported by a review performed by Chahine and collegues^22^ where bedaquiline was indicated to carry the risk of QT prolongation. As shown by the findings of this study, it is possible to assume that bedaquiline is more likely to affect the heart than the liver. As presented by the results of the study and reported by Pontali and collegues 23 bedaquiline prolongs the QT interval in the ECG and therefore, it may be harmful to the heart over a long period of time as it may result in torsades de pointes (TdP). However, the QT prolongation associated with bedaquiline can be monitored and corrected.^24^ The aminoglycosides are associated with damage to ear and kidneys when used over a long period of time, as they accumulate in the renal tubules and kanamycin affects the cochlear apparatus of the ear. Surprisingly, the current study has found that bedaquiline was more likely to cause nausea and vomiting than kanamycin (p < 0.05). It was initially thought that newer bedaquiline therapy caused less gastro-intestinal problems as compared to kanamycin and other aminoglycosides. As reported by Prasad and collegues^20^ gastrointestinal adverse effects did occur with the use of bedaquiline, therefore, it is possible that bedaquiline may be responsible for gastro-intestinal disturbances in anti-tubercular therapy. This is also supported by Gaida and collegues^25^ where the use of bedaquiline resulted in nausea and vomiting.

There were sharp increases in the number of cases of death and chest pain associated with amikacin, which was a matter of grave concern. However, there were no similar studies that reported on amikacin-associated deaths incidences to compare with the findings of this study. On the contrary, Schnippel and collegues^26^ reported that bedaquiline was associated with a reduction of mortality in patients with MDR-TB. Also, the reason for the cases of death associated with amikacin is not clearly known. However, and in corroboration of the finding of this study, Salhotra and collegues^27^ reported chest pain as one of the common adverse effects associated with bedaquiline.

It is important to note that, in primary healthcare settings, cost plays a substantial role in adherence to treatment. Bedaquiline-containing regimens are more cost-effective and have better treatment outcomes than injectable-containing regimens.^28^ This will provide an advantage to the use of bedaquiline in the healthcare system.

## Conclusion

Adverse drug reactions associated with new and old MDR-TB regimens continue to rise. Although bedaquiline causes more nausea and vomiting as compared to the aminoglycosides-based MDR-TB therapy, it is its cardiac adverse reactions that require constant monitoring. Similarly, death and chest pain cases which were associated with amikacin were worrying. Therefore, a casual link with regards to amikacin-associated death should be investigated using studies that have taken necessary confounders into consideration.

Bedaquiline will also provide a better treatment option for healthcare professionals in the primary care sector, as it is more cost-effective and has better treatment outcomes.

## Limitations of this study

This study relied solely on ADRs which were reported on VigiAccess™. Specific doses, period of therapy and the demographic details of patients were not available. Therefore, interpretation of these findings could not be extended to a causal link between specific ADRs and different anti-TB drugs. A prospective study which includes clinical follow up of patients is needed.
